# Video recording in neonatology: the need for objective measures and collaboration

**DOI:** 10.1038/s41390-024-03185-5

**Published:** 2024-04-16

**Authors:** Heidi M. Herrick, Katherine T. Wild, Morgan Hill

**Affiliations:** 1 Division of Neonatology, Department of Pediatrics, The Children’s Hospital of Philadelphia, Philadelphia, PA USA; 2 Division of Neonatology, Department of Pediatrics, Cincinnati Children’s Hospital Medical Center, University of Cincinnati College of Medicine, Cincinnati, OH USA

Video recording is a powerful, yet underutilized tool within the field of medicine, especially with regard to resuscitation and procedures. Video recording enables direct evaluation of work as done rather than work as imagined. Video recording allows the accurate assessment of timing, order, and response to interventions during resuscitations. Additionally, it can be leveraged to assess provider, team, and system performance. Within the field of neonatology, video recording has been used successfully for quality improvement initiatives, research, and education.^1–3^ To date, most studies using video recording in neonatology take place in the delivery room and primarily evaluate task performance and guideline adherence. A few studies have shifted away from this focus to evaluate non-technical skills and have adopted human factors tools to evaluate system performance.^4,5^

In the current paper, Heesters et al. describe the development of an interprofessional video review program of neonatal procedures with the aim to identify areas for improvement. Over the course of 9 months, they recorded 48 procedures and held 18 30-min interprofessional video review sessions. The review sessions were well attended with 81% of providers attending at least one session and a mean of 17 providers per session. During these sessions, providers offered their reflections and perspectives regarding the procedure. These reflections were then evaluated to identify areas for improvement by the study team, after which they were then categorized and addressed through action research cycles.

The authors took a unique approach to neonatal video review in several ways. First, the current study examined neonatal procedures including delivery room stabilization, intubations and MIST procedures, and sterile line insertion in both the delivery room and the neonatal intensive care unit. Most neonatal video review studies to date have focused solely on delivery room stabilization. Second, the authors took a deliberate approach to creating an interprofessional review program, selecting procedures that included both nurses and physicians and opening review sessions to both professions. They facilitated increased attendance of these professions by offering the sessions frequently (every 2 weeks) and both in-person and virtually. They recognized the importance of collaborative work between professions with different skill sets and areas of expertise. Creating safe opportunities for interprofessional feedback and reflection can contribute to improved comradery and culture.

Third, and arguably most importantly, the authors created structured pathways to address areas for improvement identified during video review using action research cycles of define, action, observe, and reflect. The authors developed four overarching types of interventions, (1) protocol/equipment adjustment, (2) input for research, (3) aspects of variety in care, and (4) education/training program development. The authors then classified the identified areas for improvement according to how that area might best be addressed by one of the four overarching interventions. For each of these overarching interventions, the authors developed and streamlined a pathway to efficiently address identified areas for improvement, as demonstrated by Figure 3 in their article. They identified a total of 120 areas for improvement, the majority of which were classified as aspects of variety in care.

The structured pathways the authors developed to address areas for improvement identified through video review provide a valuable template that could and should be adopted to other settings both within neonatology and within other resuscitation environments. Quality improvement projects are common in resuscitative environments, and the utilization of a structured pathway to categorize and address areas of concern has significant potential to improve efficiency and capacity of quality improvement initiatives. These pathways could be further refined and improved through the incorporation of human factors tools to objectively evaluate system performance, rather than relying solely on provider reflection and expert opinion. Human factors tools that could augment this process include formal work system analyses, hierarchical and cognitive task analyses, and flow disruption evaluation.^4,6^ These tools can identify areas of inefficiency, variation, and distractions that can negatively impact patient outcomes as well as provider and system performance. Continued adoption of these tools to neonatology and to neonatal resuscitation is a necessary next step.

Objective evaluation of performance could also be achieved by incorporating previously validated tools into the “reflect and refine” process. For example, the Behavioral Assessment Tool specifically measures behavioral performance in each of the 10 key Neonatal Resuscitation Program (NRP) behavioral skills and could provide additional objective data.^7^ This objective data would better inform the lessons learned that are used as inspiration for actions or interventions.

In addition to further adoption of human factors tools, further efforts need to focus on measuring change and sustainability of interventions over time. In the current study, the authors used the presence or absence of identified areas for improvement in repeat video review sessions to evaluate the impact of interventions and to determine if change had been sustained. Unfortunately, this metric falls short of showing true change over time. We recommend adopting formal quality improvement methodology and developing SMART Aims to address specific areas for improvement. This would not only provide objective evidence of change over time, but it would also promote the development of more formalized process measures. Furthermore, the development of neonatal resuscitation-specific quality metrics is imperative to measure the impact of interventions on patient outcomes and system performance.

Lastly, a multicenter approach to video evaluation of neonatal resuscitation and procedures is necessary to better understand universal versus site-specific areas for improvement. With few exceptions,^8^ almost all neonatal video review studies are single-center and limited by a small sample size. Collaborating across sites will increase the power of video review studies by increasing sample size and increasing generalizability of results. If common areas for improvement emerge across sites, education and guidelines may be modified to address these issues. Collaboration across sites will also facilitate improved dissemination of knowledge and reciprocal learning.
